# UCP2 Modulates Cardioprotective Effects of Ru360 in Isolated Cardiomyocytes during Ischemia

**DOI:** 10.3390/ph8030474

**Published:** 2015-08-04

**Authors:** Lukas J. Motloch, Sara Reda, Martin Wolny, Uta C. Hoppe

**Affiliations:** Department of Internal Medicine II, Paracelsus Medical University, Salzburg 5020, Austria; E-Mails: sara.reda@pmu.ac.at (S.R.); martinwolny@yahoo.de (M.W.); u.hoppe@salk.at (U.C.H.)

**Keywords:** mitochondrial calcium uniporter, ruthenium, UCP2, ischemia, cardiomyocytes, cardioprotection

## Abstract

*Introduction:* Ruthenium 360 (Ru360) has been shown to induce cardioprotective mechanisms in perfused hearts. The agent is a specific blocker of the main cardiac mitochondrial uptake mechanism, the mitochondrial calcium uniporter (MCU). UCP2, a mitochondrial membrane protein, which influences cardiac ROS formation was reported to interact with the MCU. *Methods*: To prove whether Ru360 affects ischemic cell injury on the singular cell level, cell viability (CV) in isolated cardiomyocytes from wild type mice (WT) was measured in a model of pelleting hypoxia (PH). To explore a possible influence of UCP2 on cellular survival, as well as on Ru360 function, cardiomyocytes from UCP2^−/−^ mice were investigated. *Results*: During PH, Ru360 significantly improved CV in WT cardiomyocytes (Control 26.32% ± 1.58% *vs.* PH 13.60% ± 1.20% *vs.* PH+Ru360 19.98% ± 0.98%, *n* = 6; *p* < 0.05). No differences in the rate of apoptosis were observed in UCP2^−/−^ *vs.* WT. In UCP2^−/−^ cardiomyocytes, Ru360 reduced the rate of cell death. However, the effect was less pronounced compared to WT cardiomyocytes. *Conclusion*: Ru360 significantly reduces hypoxic cell injury by preventing single cell apoptosis in WT cardiomyoctes. UCP2 does not affect cell survival in hypoxic cardiomyocytes, but it might modulate cardioprotective effects of Ru360 during ischemia.

## 1. Introduction

Myocardial ischemia is one of the major causes of morbidity and mortality in Western nations [1]. Especially prolonged periods of ischemia can cause cardiac tissue injury and cell death. Although, during ischemia, a large portion of cells die due to necrosis, the cognate but more well-regulated mechanism of apoptosis is known to increase further cell loss and surrounding tissue damage [2,3,4].

Mitochondrial calcium handling is a key regulator of several important processes in cellular physiology. It contributes to the spatial and temporal profile of intracellular calcium signaling [5,6], the production of ATP [7,8] and the synthesis of reactive oxygen species (ROS), which are generated during oxidative stress situations like ischemia [9]. Increased mitochondrial calcium uptake, as well as elevated cytosolic ROS concentrations, are able to induce the formation of the mitochondrial transition pore (mPTP). Thus, increased mitochondrial calcium uptake provokes the mechanism of apoptosis and triggers cells death causing enhanced tissue damage in oxidative stress situations like myocardial ischemia [5,10,11]. Indeed, in first clinical trials, inhibition of the mPTP during myocardial infarction presented promising results [12]. Therefore, one could speculate that a reduction of mitochondrial calcium uptake during cardiac ischemia may decrease cardiac apoptosis rate and display cardioprotective effects.

Mitochondrial calcium influx is thought to be mostly mediated by the mitochondrial calcium uniporter (MCU), which is located in the inner mitochondrial membrane [5,11,13,14]. The MCU is known to be blocked by Ruthenium Red (RuRed) or by its more potent derivative Ruthenium360 (Ru360) [13,14,15,16]. In fact in perfused rat hearts both pharmacological agents were able to reduce ischemia reperfusion (I/R) injury by improving oxygen efficiency, increasing the recovery of the left ventricular contractility and reducing the incidence of post reperfusion arrhythmias [17,18,19,20,21]. Some authors suggested that these described cardiorotective pharmacological effects may be caused by an MCU mediated ischemic preconditioning [21]. However, whether these observations were based on the prevention of apoptosis on the level of the singular cardiomyocyte, which would exclude further possible influences like cardiomyocyte crosstalk through gap junctions or the interaction of cardiomyocytes with adjacent cell types (fibroblasts, endothelial cells *etc.*) remains unclear.

UCP2 is a mitochondrial membrane protein, which belongs to a superfamily of mitochondrial ion transporters [22]. In the heart, the protein was proven to suppress ROS production [22,23]. Furthermore, it has been shown to modulate mitochondrial calcium uptake [24,25,26], which is probably induced by an interaction between UCP2 and the MCU [24,27,28]. Therefore, one might speculate that during ischemia, UCP2 influences cardioprotective implication of MCU inhibition. Furthermore, whether the protein affects the rate of apoptosis during ischemia remains unclear.

Therefore, in order to explore potential cardioprotective consequences of MCU inhibition on the level of a singular cardiomyocyte, in this study, we used a model of pelleting hypoxia (PH) [29] in isolated cardiac myocytes from wild type (WT) mice. Furthermore, in order to evaluate possible influences of UCP2 on cellular survival during hypoxia, cardiomyocytes from UCP2^−/−^ mice were investigated.

## 2. Results

**Figure 1:**
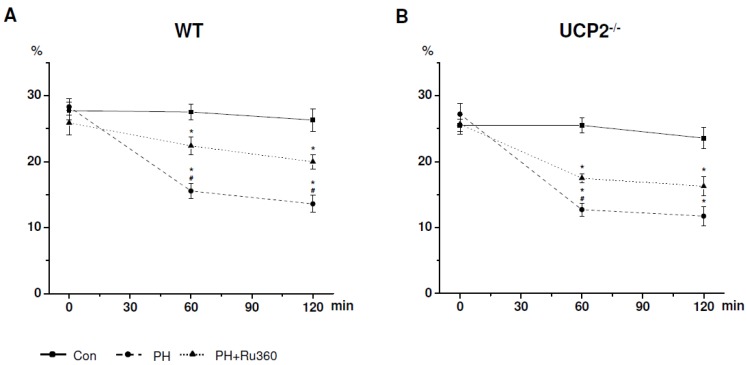
Total cell viability after 0, 60 and 120 min (**A**) in WT and (**B**) in UCP2^−/−^ cardiomyocytes. Con = cells stored in control solution; PH = pelleting hypoxia alone; PH+Ru360 = pelleting hypoxia previously incubated with Ru360 (10 µM); * *p* < 0.05 *vs.* Con; **^#^** *p* < 0.05 *vs.* PH.

Using the Langendorff isolation technique, cell viability (CV) after enzymatic digestion was similar in WT and UCP2^−/−^ (CV: WT 27.30% ± 0.85%, *n* = 6 *vs.* UCP2^−/−^ 26.12% ± 0.75%, *n* = 5; *p* > 0.05; Figure 1). Stored in a physiological solution (Con), isolated cardiomyocytes from both animal models were able to survive for 120 min (Figure 1, Table 1 and Table 2).

**Table 1:** Cell viability in WT.

WT (*n* = 6)	Cell Viability (%)		
	0 min	60 min	120 min
Control	27.72 ± 1.25	27.51 ± 1.14	26.32 ± 1.58
PH	28.31 ± 1.13	15.55 ± 1.06 *	13.60 ± 1.20 *
PH+Ru360	25.88 ± 1.72	22.39 ± 1.21 *^#^	19.98 ± 0.98 *^#^

PH = pelleting hypoxia; PH+Ru360 = pelleting hypoxia previously incubated with Ru360 (10 µM); * *p* ˂ 0.05 *vs.* control; **^#^** *p* ˂ 0.05 *vs.* PH.

### 2.1. CV after Induction of Ischemia in WT Cardiomyocytes

In WT cardiomyocytes, PH alone significantly reduced cellular survival after 60 (Figure 1a, Table 1) and 120 min (Figure 1a, Table 1). Cell death during hypoxia could be reduced using Ru360 (10 µM). Compared to hypoxia alone, myocytes incubated with Ru360 presented a significant improvement in CV after 60 (Figure 1a, Table 1) and after 120 min (Figure 1a, Table 1), indicating that during ischemic stress Ru360 displays cardioprotective effects in singular WT cardiomyocytes.

### 2.2. CV after Induction of Ischemia in UCP2^−/−^ Cardiomyocytes

Similar to WT, PH alone significantly decreased cell survival of UCP2^−/−^ cardiomyocytes after 60 (Figure 1b, Table 2) and after 120 min (Figure 1b, Table 2). In UCP2^−/−^, treatment of cardiomyocytes with Ru360 significantly increased CV during 60 min of hypoxia (Figure 1b, Table 2). Following 120 min of hypoxia the pharmacological agent showed a trend towards an elevation in cardiomyocytes survival without reaching statistical significance.

**Table 2:** Cell viability in UCP2^−/−^.

UCP2^−/−^ (*n* = 5)	Cell Viability (%)		
	0 min	0 min	0 min
Control	25.50 ± 0.77	25.50 ± 0.97	23.56 ± 1.37
PH	27.19 ± 1.33	12.71 ± 0.83 *	11.73 ± 1.23 *
PH+Ru360	25.67 ± 1.26	17.49 ± 0.58 *^#^	16.26 ± 1.25 *

PH = pelleting hypoxia; PH+Ru360 = pelleting hypoxia previously incubated with Ru360 (10 µM); * *p* ˂ 0.05 *vs.* control; **^#^** *p* ˂ 0.05 *vs.* PH.

### 2.3. RRCD in WT vs. UCP2^−/−^ Cardiomyocytes

**Figure 2:**
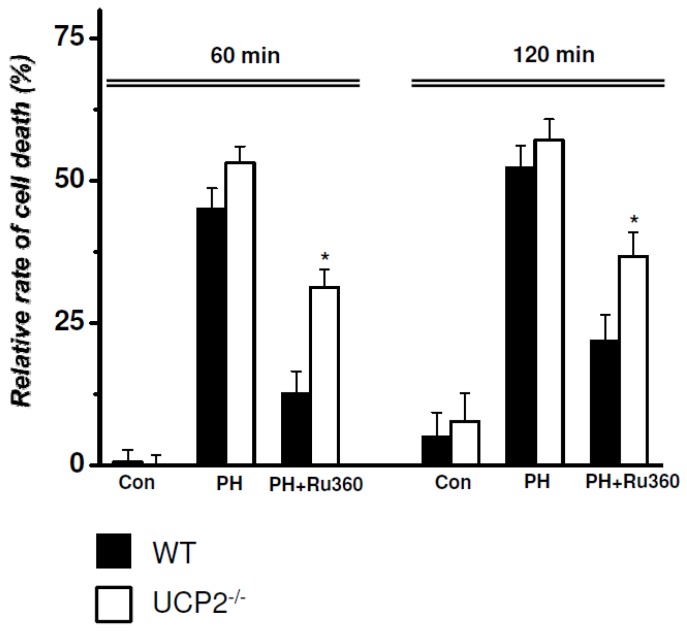
Relative rate of cell death (RRCD) defined as the percent of the difference in CV at 0 min and at X min normalized to CV at 0 min in WT *vs.* UCP2^−/−^ cardiomyocytes after 0, 60 and 120 min. Con = cells stored in control solution; PH = pelleting hypoxia; PH+Ru360 = pelleting hypoxia previously incubated with Ru360 (10 µM); * *p* < 0.05 *vs.* WT.

To further evaluate whether UCP2 may modulate ischemic injury and/or cardioprotective effects of Ru360, we calculated the relative rate of cell death (RRCD defined as the percent of the difference in CV at 0 min and at X min normalized to CV at 0 min) in WT and UCP2^−/−^. Hypoxia alone induced a similar RRCD in both animal models after 60 (Figure 2, Table 3) and after 120 min (Figure 2, Table 3), indicating that, in singular cardiomyocytes, UCP2 does not affect cell injury during ischemia.

**Table 3:** Relative rate of cell death.

WT (*n* = 6) *vs.* UCP2^−/−^ (*n* = 5)		Relative Rate of Cell Death (%)	
		60 min	120 min
**Control**	WT	0.54 ± 2.00	5.04 ± 3.95
	UCP2^-/-^	0.12 ± 1.41	7.70 ± 4.27
**PH**	WT	44.98 ± 3.45	52.25 ± 3.56
	UCP2^-/-^	53.12 ± 2.43	57.10 ± 3.12
**PH+Ru360**	WT	12.64 ± 3.58	21.74 ± 4.26
	UCP2^-/-^	31.30 ± 2.67 *	36.68 ± 3.67 *

PH = pelleting hypoxia; PH+Ru360 = pelleting hypoxia previously incubated with Ru360 (10 µM); * *p* ˂ 0.05 *vs.* WT.

However, compared to WT cells incubated with Ru360, in treated UCP2^−/−^ cardiomyocytes, RRCD was significantly increased after 60 (Figure 2, Table 3) and 120 min (Figure 2, Table 3), respectively, suggesting a diminished cardioprotective function of the drug in the absence of the mitochondrial protein.

## 3. Discussion

In this study we used a model of hypoxia in isolated cardiomyocytes. Our experiments were able to confirm cardioprotective effects of the specific MCU blocker Ru360. Previous trials in perfused rat hearts had already suggested beneficial implications of MCU inhibition during I/R [17,18,19,20,21]. However, our results extend these observations. Our study indicates that Ru360 might induce cardioprotective effects by decreasing the rate of apoptosis directly on the singular cell level in the absence of further possible influences like cardiomyocyte crosstalk through gap junctions or the interaction of cardiomyocytes with adjacent cell types (fibroblasts, endothelial cells, *etc.*). Thus, it is able to reduce ischemic injury.

Since Ru360 is known to be a specific inhibitor of the MCU [13,14,15,16], one could speculate that by decreasing MCU calcium transport activity during ischemia, Ru360 might prevent mPTP formation and therefore obviate the initiation of programmed cell death in singular cardiomyocytes. Thus, we assume that inhibition of MCU using Ru360 seems to be a promising approach for the therapy of ischemic heart injury.

Furthermore, using a murine knock out model, in this study we investigated whether the mitochondrial protein UCP2 may influence hypoxic injury in isolated cardiomyocytes. UCP2 was shown to influence mitochondrial calcium uptake [24,25,26]. Some studies suggested an interaction of this protein with the MCU [24,27,28] and furthermore presented a UCP2 dependent inhibition of the uniporter by RuRed in UCP2^−/−^ mice [24]. In addition, UCP2 was proven to prevent ROS formation and, thus, it was proposed to have cardioprotective influences [23]. Compared to WT, our experiments did not reveal a significant impact of UCP2 on cellular apoptosis after the induction of PH alone. However, UCP2^−/−^ cardiomyocytes showed to be less sensitive to Ru360 cardioprotection. This observation indicates a potential role of the protein during drug channel interaction in the case of hypoxic stress and supports previous results obtained in UCP2^−/−^ mice [24].

## 4. Experimental Section

The present study was performed with approval of the local Ethical Committee and conforms to the Guide for the Care and Use of Laboratory Animals published by the US National Institutes of Health (NIH publication No. 85-23, revised 1996).

### 4.1. Animals

The mouse strain B6.129S4-Ucp2^tm1Lowl^/J (UCP2^−/−^) was purchased from Charles River Laboratories, Research Models and Services (Sulzfeld, Germany) [30]. The identical background strain was used as control (WT). The animals used in this study were male between 8 and 12 weeks of age.

### 4.2. Preparation of Murine Cardiomyocytes

Hearts were obtained from WT or UCP2^−/−^ mice, and single ventricular myocytes were isolated from murine hearts by enzymatic digestion using a Langendorff perfusion system, as previously described [31,32]. Experiments were only continued if CV after enzymatic digestion reached at least 20%.

### 4.3. Pelleting Hypoxia

CV experiments during pelleting hypoxia were performed as previously described [29,33]. Isolated myocytes of C57BL/6J mice were aliquoted into three groups: control (Con), pelleting hypoxia alone (PH) and PH previously (15 min before PH) incubated with Ru360 (10 µM, Sigma Aldrich, Munich, Germany; PH+Ru360). PH was induced by pelleting the cells in a hypoxic solution, pH adjusted to 6.5, and sealing the pellet with a layer of mineral oil, using a protocol previously reported [33]. Solutions contained: Control: NaCl 125 mM, KCl 5.4 mM, NaH_2_PO_4_ 1.2 mM, MgCl_2_ 0.5 mM, HEPES 20 mM, glucose 15 mM, taurine 5 mM, CaCl_2_ 1 mM, creatine 2.5 mM, BSA 0.1%, pH 7.4, 100% O_2_, 310 mOsm/L. Hypoxic: NaCl 119 mM, KCl 5.4 mM, MgSO_4_ 1.3 mM, NaH_2_PO_4_ 1.2 mM, HEPES 5 mM, MgCl_2_ 0.5 mM, CaCl_2_ 0.9 mM, Na-lactate 20 mM, BSA 0.1%, 310 mOsm/L, pH 6.5. At 0, 60, and 120 min, 10 μL samples of cardiomyocytes from each group were resuspended for 3–5 min in control (at 0 min) or hypoosmolar solution (at 60 and 120 min, final osmolarity: 250 mOsm/L with NaCl reduced to 88 mM), with 0.5% trypan blue (Sigma Aldrich) added.

Images of cell morphology were recorded at 100 × magnification on an Axiovert 200 microscope (Zeiss, Göttingen, Germany) for subsequent off-line analysis by an examiner blinded to the group. A total of 200 cells or more were counted per sample. CV was quantified as the percent of rod-shaped, unstained cells over all cells. Relative rate of cell death (RRCD) was defined as the percent of the difference in CV at 0 min and at X min normalized to CV at 0 min.

### 4.4. Statistical Analysis

Pooled data are presented as mean ± SEM. Comparisons of CV between groups were performed with one-way ANOVA followed by Bonferroni correction. Differences in relative rate of cell death in WT *vs.* UCP2^−/−^ were compared using unpaired t-test. Probability values of *p* < 0.05 were regarded statistically significant.

## 5. Conclusions

In conclusion, our results suggest that Ru360 prevents hypoxic injury by decreasing the rate of apoptosis in singular cardiomyocytes. Experiments performed in UCP2^−/−^ cardiomyocytes did not prove an impact of UCP2 on cellular survival during hypoxic stress. However, our observations suggest that UCP2 modulates cardioprotective effects of Ru360 during ischemia.
